# Rotarod training reverses corticosterone-induced motor deficits via oligodendrocyte lineage cell modulation

**DOI:** 10.4103/NRR.NRR-D-24-00448

**Published:** 2025-07-05

**Authors:** Jada Chia-Di Lee, Benson Wui-Man Lau, Suk-Yu Yau, Joseph Wai-Hin Leung, Harmony Kai-Hei Wong, Dalinda Isabel Sanchez Vidana, Tatia M.C. Lee, Wu-Tian Wu, Kwok-Fai So

**Affiliations:** 1 School of Biomedical Sciences, Li Ka Shing Faculty of Medicine, The University of Hong Kong, Hong Kong Special Administrative Region, China; 2 Department of Rehabilitation Sciences, Faculty of Health and Social Sciences, The Hong Kong Polytechnic University, Hong Kong Special Administrative Region, China; 3 The State Key Laboratory of Brain and Cognitive Sciences, The University of Hong Kong, Hong Kong Special Administrative Region, China; 4 Laboratory of Neuropsychology, The University of Hong Kong, Hong Kong Special Administrative Region, China; 5 Laboratory of Cognitive Affective Neuroscience, The University of Hong Kong, Hong Kong Special Administrative Region, China; 6 Guandong-Hong Kong-Macau Institute of CNS Regeneration (GHMICR), Jinan University, Guangzhou, Guangdong Province, China; 7 Department of Ophthalmology, Li Ka Shing Faculty of Medicine, The University of Hong Kong, Hong Kong Special Administrative Region, China

**Keywords:** corticosterone-induced stress, exercise, major depressive disorder, motor cortex, motor deficits, motor training, myelination, oligodendrocyte lineage cells, oligodendrocyte precursor cells, psychomotor retardation

## Abstract

Adult-born oligodendrocytes are continuously produced in the brains of rodents. The functional role of these cells has been linked to the motor-related activities of healthy animals and is vital for acquiring new motor skills. However, the relationship between these cells and the control of motor-related activities has not been investigated in pathological conditions. Therefore, the aim of this study is to investigate the role of oligodendrocytes in depression-related motor deficits and the effects of training. Psychomotor retardation is a key symptom of depression. Consistent with the impairments observed in rodent motor performance, the proliferation and activation of adult-born oligodendrocytes are altered in a corticosterone-induced stress paradigm. Therapeutic rotarod training can alleviate these symptoms by reversing the aforementioned changes. Notably, these alterations are particularly pronounced in layer I of the motor cortex. Thus, this study provides evidence of the potential functional involvement of adult-born oligodendrocytes in the motor impairments observed in the depressed animals. Additionally, it offers preliminary results for further investigation into layer I of the motor cortex in relation to these pathological conditions.

## Graphical abstract

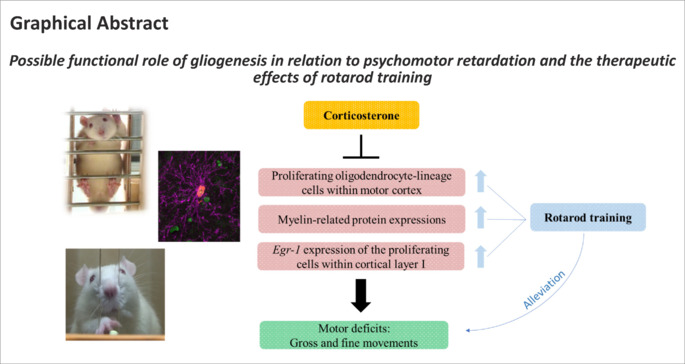

## Introduction

Psychomotor retardation (PMR) is recognized as one of the core symptoms of major depressive disorder. The motor characteristics of PMR observed in depressed patients include changes in gross activity, fine motor movements, and increased response latency (Pier et al., 2004). Despite being acknowledged for a long time in individuals with depression, the underlying neurobiology of this symptom remains poorly understood.

Recent studies have linked structural alterations in regions of the motor system, such as the motor cortex, basal ganglia, and dorsolateral prefrontal cortex, to the symptom of psychomotor retardation (PMR) in depression (Buyukdura et al., 2011; Bracht et al., 2012; Ma et al., 2022). Numerous studies have indicated that the integrity of white matter connecting these motor system regions is altered in depressed patients compared to healthy controls (Bracht et al., 2012; Zhang et al., 2018; Carceller-Sindreu et al., 2019). These alterations observed in depressed individuals are thought to be associated with a reduced number of oligodendrocytes and myelination in the brain (Tham et al., 2011; Walther et al., 2012).

Oligodendrocyte precursor cells (OPCs), also known as NG2 cells (chondroitin sulfate proteoglycan neuron-glia antigen 2), are glial cells that differentiate into myelinating oligodendrocytes during both developmental and postnatal stages (Trotter et al., 2010). It has since been discovered that these OPCs can undergo dynamic development throughout adulthood and can be influenced by various behavioral experiences. For example, social deprivation has been shown to reduce adult myelination in the rodent prefrontal cortex, a region involved in cognitive functions and emotions (Liu et al., 2012). In contrast, an increased number of myelinating oligodendrocytes, specifically 2′,3′-cyclic nucleotide 3′-phosphodiesterase (CNPase) positive cells, have been observed in the corpus callosum of rodents exposed to an enriched environment. Moreover, this enhancement in myelination has been suggested to correlate with improved spatial learning (Zhao et al., 2011).

The aforementioned behavioral experiences also significantly influence stress modulation in depression. The alteration of hippocampal neurogenesis due to stress has been hypothesized as a potential pathophysiology of depression (Jacobs et al., 2000). For instance, the promotion of neurogenesis through positive factors, including environmental enrichment and physical exercise, could alleviate depressive symptoms in relation to cognitive functions and emotions (Jha et al., 2011; Sakalem et al., 2017). In addition to alleviating depressive symptoms by enhancing neurogenesis, physical exercise can also increase the population of OPCs in the adult brain, including regions such as the prefrontal cortex (Mandyam et al., 2007), amygdala (Ehninger et al., 2011), the cingulate cortex (Ehninger and Kempermann, 2003), and the motor cortex (Simon et al., 2011) under normal conditions. The increase of OPCs in the substantia nigra, a region associated with motor control, has been linked to improved motor functions in a rat model of Parkinson’s disease (Steiner et al., 2006). Another study by McKenzie et al. (2014) demonstrated that active myelination in the adult brain is necessary during motor skill learning on a complex wheel. These findings provide possible evidence that adult-born oligodendrocyte lineage cells and myelination influence motor-related activities in rodents. However, the potential involvement of oligodendrocytes and myelination in causing the pathological symptom of psychomotor retardation in a depressive animal model has yet to be demonstrated (Zhou et al., 2021). Additionally, little is known about whether a positive factor, such as motor training, can be utilized as a potential therapeutic approach to alleviate PMR in depression by modulating the population of oligodendrocyte lineage cells.

The aim of this study was to investigate whether alterations in oligodendrocyte development contribute to psychomotor retardation observed in depression and to determine whether enhancing oligodendrocyte populations through motor training can mitigate these symptoms. Given the gap in understanding the role of oligodendrocytes in motor control under pathological conditions, we hypothesized that depressive states impair oligodendrocyte function in the motor cortex, contributing to psychomotor retardation, and that targeted motor training could activate oligodendrocytes to alleviate this symptom. This study seeks to elucidate the functional involvement of oligodendrocyte lineage cells in motor deficits associated with depression, providing insights into potential therapeutic strategies.

## Methods

The animal experiments were approved by the Committee on the Use of Live Animals in Teaching and Research of Li Ka Shing Faculty of Medicine at The University of Hong Kong (Ref: CULATR3492-14, November 10, 2014) and reported according to the Animal Research: Reporting of *In Vivo* Experiments (ARRIVE) guidelines (Percie du Sert et al., 2020). All efforts were made to minimize animal suffering and the number of animals used.

### Animals and 14-day treatments

Adult male Sprague-Dawley rats with normal microbiological status (*n* = 24, Laboratory Animal Unit, The University of Hong Kong, China) weighing 250 ± 20 g were used for the experiment. Male rats were used because they exhibited more consistent immobility times in the forced swim test, reducing variability in depressive-like behavior assessments (Ma et al., 2019). The rats were housed individually and kept under a 12/12 light-dark cycle at 25°C and 40%–60% humidity, with ad libitum access to food and water. After a two-day adaptation period, the rats (6 rats per group) randomly received either subcutaneous injections of a vehicle (sesame oil) or 40 mg/kg of corticosterone (CORT) continuously for 14 days. Sesame oil alone was injected as a sham control to assess the effects of the vehicle (Skorzewska et al., 2006). The corticosterone, at a dosage of 40 mg/kg (Cat# C2505, Sigma-Aldrich, St. Louis, MO, USA), was freshly prepared before use by vortexing with sesame oil (Cat# S3547, Sigma-Aldrich) and sonicating for 60 minutes. The prepared corticosterone was injected subcutaneously daily into the back of the neck (Hellsten et al., 2002). Before each injection, the emulsion was briefly vortexed to ensure homogeneity (Hellsten et al., 2002). The injections were administered subcutaneously in the neck region at a dose of 40 mg/kg daily (Hellsten et al., 2002). Rats in the vehicle control group were injected daily with only sesame oil as a vehicle treatment.

The rats underwent a 14-day drug treatment and were randomly divided into four groups (*n* = 6 rats per group): 1) Vehicle-treated without motor training (V_N); 2) Vehicle-treated with motor training (V_T); 3) Corticosterone-treated without motor training (CORT_N); and 4) Corticosterone-treated with motor training (CORT_T). The animals assigned to motor training were placed on a rotating rotarod (Huaibei Zhenghua Biologic Apparatus Facilities Limited Company, Huaibei, Anhui Province, China) that rotated at a constant speed of 10 revolutions per minute for 10 minutes each day. For those assigned to the group without rotarod training, they were placed on the rotarod for 10 minutes each day, but the device was not activated.

### Animal behavioral tests

#### Forced swimming test

The forced swimming test (FST) was conducted by placing a rat into a cylindrical tank with a height of 60 cm, filled with tap water at 23°C, with the water level set at 30 cm (Brenes Sáenz et al., 2006). The rats were exposed to the tank on two occasions. After 14 days of drug treatment, the rats were placed into the cylindrical tank filled with water for 15 minutes as a pre-test session. They were then placed into the tank again 24 hours later for 5 minutes as the test session. The 5-minute test was videotaped using a SONY color digital camera and recorder (SONY, Hong Kong Special Administrative Region, China). The behavior scoring, specifically the immobility time characterized by minimal action in the water during the 5-minute test, was determined by a single rater who was blind to the animal treatments. Data were presented as average immobility time ± SEM and were compared among the four treatment groups.

#### Sucrose preference test

The sucrose preference test (SPT) was conducted in this study to assess the anhedonic state induced by stress in rodents (Brenes Sáenz et al., 2006). Each rat was individually housed and given access to food, as well as two bottles, one containing tap water and the other containing a 1% sucrose solution. The bottles were weighed before the start of the test and then reweighed at 12-hour intervals, for a total of two measurements. The data were presented as the average percentage of sucrose consumed relative to the total fluid intake (the sum of tap water and sucrose solution consumed).

#### Ladder beam walking test

The ladder beam walking test (LBWT) was used to evaluate the hindlimb functions of the animals by examining the accuracy of their hindlimb placement while crossing a 100-cm-long runway (Metz and Whishaw, 2002). The runway consisted of irregularly spaced metal rungs with intervals ranging from 3 cm to 5 cm. It was elevated 55 cm above the ground, with the home cage containing the rats’ littermates positioned at the end. A digital video camera was mounted underneath the ladder to record the rats’ walking process. If a hindlimb fell through the metal rungs, it was counted as an error, and the number of errors made by each hindlimb was analyzed from the recordings. The rats underwent a pre-training period of 2 days prior to the 14-day treatment, during which they needed to cross the ladder five times each day. Only the results obtained on the second day were used for baseline analysis. Following the 14-day treatment, the rats were allowed to access the ladder beam again for five trials. Data were presented as the average number of foot faults for each rat across the five trials, reported as ± SEM, and the experiment was conducted in a blinded manner.

#### Skilled reaching test

The skilled reaching test (SRT) was applied in this study to determine the forelimb performance of the animals (Nica et al., 2018). Food deprivation was required 18 hours before each session of skilled reaching training and test. The SRT involved placing the rats in a Plexiglas box measuring 45 cm × 40 cm and 13.1 cm wide before the commencement of the drug treatment (Nica et al., 2018). A long shelf extended 6 cm from the front wall at a height of 4 cm. In the center of the front wall of the skilled reaching box, there was a 1-cm-wide vertical opening that allowed the rats to access food pellets (45-mg banana-flavored precision pellets, Bioserve, Frenchtown, NJ, USA) through the slit. The training procedure lasted for 10 days prior to the 14-day treatment period. On the final day of the pre-training session, each rat was required to complete three trials, reaching 10 pellets per trial before the start of the 14-day treatment. After the drug treatment, the rats were placed back in the skilled reaching box to perform the reaching task for three trials, attempting to reach 10 pellets per trial in order to evaluate changes in forelimb function following drug treatment. These procedures were videotaped for further analysis. Data were presented as the average percentage of successful attempts out of the 10 pellets provided, expressed as ± SEM. A “successful reach” was defined as an instance where the rats could grasp the food pellet on their first attempt and subsequently deposit it into their mouths. Instances where the rats made multiple attempts to grasp the food but were unable to successfully put the food pellets into their mouths were counted as “misses” and were not included in the tally of “successful reaches.”

### Immunohistochemistry

The rats were deeply anesthetized with sodium pentobarbital (Merk, Hong Kong Special Administrative Region, China) administered intraperitoneally at a dose of 200 mg/kg and perfused with 0.9% normal saline, followed by 4% paraformaldehyde. The brains were then extracted and post-fixed overnight in 4% paraformaldehyde at 4°C. They were cryoprotected in 30% sucrose in 0.1 M phosphate buffer for sectioning until they sank. Coronal sections were cut to a thickness of 30 µm; these sections were collected and stored in a 12-well plate with cryoprotectant at 4°C until immunostaining. The stored frozen sections were affixed to gelatin-coated slides and air-dried overnight. Antigen retrieval was performed by incubating the slides in citric acid (pH 6.0) (Cat# 13735, USB, Affymetrix, Cleveland, OH, USA) at 90°C for 30 minutes, after which the sections were washed with 0.01 M phosphate-buffered saline (PBS). The sections were then incubated in 1 M hydrochloric acid for 30 minutes at 37°C, followed by treatment with borax buffer (pH 8.0) for 15 minutes to achieve neutralization. The sections were incubated overnight at room temperature with primary antibodies: mouse anti-bromodeoxyuridine (BrdU; 1:1000, Cat# ab6326, Abcam, Hong Kong Special Administrative Region, China), mouse anti-NG2 (1:150, Cat# ab5320, Millipore, Hong Kong Special Administrative Region, China), mouse anti-Olig2 (1:200, Cat# ab9610, Millipore), mouse-anti Iba-1 (1:200, Cat# 019-19741, Wako, Osaka, Japan), mouse anti-GFAP (1:500, Cat# Z0334, Dako, Hong Kong Special Administrative Region, China), mouse anti-DCX (1:200, Cat# 4604, Cell Signaling Technology, Danvers, MA, USA), mouse anti-CNPase (1:200, Cat# MAB326, Millipore), and mouse anti-egr-1 (1:200, Cat# 133695, Abcam). Incubation with the primary antibody was done at 4°C overnight on a rocking platform. The sections were washed with PBS and incubated with secondary goat anti-mouse fluorescence antibodies for 2 hours (Alexa Fluor 488 or 568, Thermo Fisher Scientific, Hong Kong Special Administrative Region, China). Incubation with the secondary antibody was done at 25°C for 2 hours on a rocking platform. Finally, the sections were covered with a coverslip using a mounting medium (Invitrogen, Camarillo, CA, USA) and stored in the dark.

### Cell quantification

Four coronal planes, located 0.30 mm, 0.80 mm, 1.80 mm, and 2.33 mm posterior to bregma, were selected from each animal for outlining the motor cortex, as previously described by Anderson et al. (2002). The motor cortex was further subdivided into layers I, II–III, and V–VI, with the layers outlined using the StereoInvestigator software (MicroBrightField Bioscience, Williston, VT, USA) at a 4× objective. The number of BrdU-positive cells in layers I, II–III, and V–VI was estimated using an optical fractionator, which employed a counting frame size of 60 μm × 60 μm that was systematically random-sampled across the outlined layers. A guard zone height of 5 μm and a dissector height of 10 μm were utilized. Additionally, layer I of the piriform cortex in the four coronal planes was outlined using the StereoInvestigator method as described above. Results were presented as the number of cells per section ± SEM. The cell quantification process was conducted in a blinded manner, ensuring that the investigator was unaware of the treatment group of the animals.

To further identify the BrdU-positive cells in layer I of the motor cortex, BrdU-positive cells were double-labeled with oligodendrocyte markers (NG2, Olig2), microglial marker (Iba-1), astrocyte marker (GFAP), and neuronal lineage marker (DCX). At least 20 BrdU-positive cells were randomly selected within the outlined layer I in each coronal plane (0.30 mm, 0.80 mm, 1.80 mm, and 2.33 mm posterior to bregma). Data were presented as the averaged percentage of BrdU cells expressing either the oligodendrocyte, microglia, astrocyte, or neuronal marker ± SEM.

### Western blotting

Proteins were extracted from tissue samples using a lysis buffer consisting of Radioimmunoprecipitation Assay (RIPA) buffer (Cat# 20-188, Millipore), supplemented with 1 mM phenylmethylsulfonyl fluoride (Cat# 10837091001, Sigma-Aldrich), protease inhibitors (Cat# 539137-10vlcn, Millipore), and phosphatase inhibitors (Cat# 539131, Calbiochem). The brain tissue was homogenized in the lysis buffer and incubated on ice for 30 minutes to facilitate protein solubilization. Following this, the homogenate was centrifuged at 12,000 × *g* for 10 minutes at 4°C to pellet cellular debris. The supernatant containing the extracted proteins was collected and stored at –80°C until further analysis. Prior to Western blot analysis, protein concentration in the samples was quantified using the BCA Protein Assay Kit (Thermo Fisher Scientific), following the manufacturer’s instructions to ensure equal protein loading across samples in subsequent gel electrophoresis. Equal amounts of protein (10–15 µg per lane) were mixed with 4× Laemmli sample buffer (Cat# 161-0737, Bio-Rad, Hong Kong Special Administrative Region, China) and heated at 95°C for 5 minutes to denature the proteins. The samples were loaded into the wells of a 10% polyacrylamide gel prepared using the Mini-PROTEAN Tetra System (Bio-Rad). A protein ladder (e.g., PageRuler Prestained Protein Ladder, Thermo Fisher Scientific) was included in one lane for size estimation. The gel was run at 100 V before transfer. After electrophoresis, proteins were transferred from the gel to a nitrocellulose membrane (Cat# 162-0112, Bio-Rad) using a wet transfer method. The transfer was conducted at 100V for 1 hour at 4°C, using a transfer buffer containing 25 mM Tris, 192 mM glycine, and 20% methanol. The blot was incubated in 5% non-fat dry milk in Tris-buffered saline with Tween 20 (TBST) for 1 hour at room temperature. After blocking, the membrane was washed three times with TBST for 5 minutes. The membrane was then incubated overnight at 4°C with the primary mouse anti-CNPase antibody (1:1000, Cat# MAB 326, Millipore). Following incubation, the membrane was washed again three times with TBST for 5 minutes. Subsequently, the membrane was incubated with horseradish peroxidase (HRP)–conjugated secondary antibody specific to mouse IgG (1:200, Cat# P0447, Dako) for 1 hour at room temperature. The membrane was washed three times with TBST for 5 minutes. The immunoreactive bands were visualized using a chemiluminescent HRP detection reagent (Cat# WBLUF0100, Millipore). After incubation for 5 minutes, the detection was performed using a chemiluminescence imaging system (Bio-Rad). To ensure equal protein loading across different samples, membranes were subsequently probed with a rabbit antibody against GAPDH (1:5000, Cat# A5316, Sigma-Aldrich). The corresponding goat HRP-conjugated secondary antibody to rabbit (1:5000, Cat# P0448, Dako) was applied following the same washing and detection protocol as described above. Densitometric analysis of the Western blot protein bands was conducted using ImageJ software (version 1.51, National Institute of Health, Bethesda, MD, USA). The intensity of the bands corresponding to CNPase was normalized to the intensity of the GAPDH band to account for variation in loading across the samples.

### Statistical analysis

No statistical methods were used to predetermine sample sizes; however, our sample sizes are similar to those reported in previous publications (Sánchez-Vidaña et al., 2016， 2019; Chan et al., 2017). Statistical analysis was conducted to assess the effects of corticosterone and rotarod training on various behavioral outcomes, including measures of depression-like behavior, anhedonic state, ladder beam walking, and skilled reaching, using one-way analysis of variance followed by the least significant difference post hoc test, with a sample size of six animals per group (*n* = 6). Furthermore, the impact of these treatments on BrdU cells co-expressing oligodendrocyte markers, such as NG2, Oligo2, Iba-1, GFAP, and DCX, was evaluated. Data were presented as averaged percentages of BrdU cells expressing each marker, with a total of four sections per animal analyzed for this purpose. In each section, 20 BrdU-positive cells were randomly selected to ensure robust data collection. Additionally, the expression of CNPase was measured through western blot analysis, employing the same statistical framework of one-way analysis of variance and the least significant difference *post hoc* test for comparison among treatment groups, with one tissue sample analyzed per rat in each group. A statistically significant difference was defined as *P* < 0.05, and all data are expressed as mean ± SEM.

## Results

### Animal model and behavioral assessments

**Figure 1:**
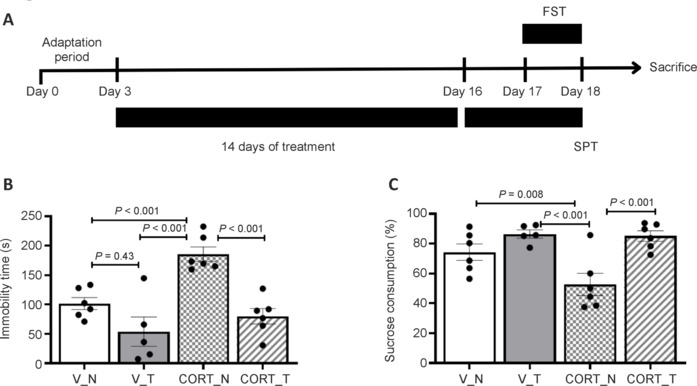
Induction of depression-like behaviors by corticosterone and therapeutic role of rotarod training on animal behaviors. (A) The experimental timeline shows the sequence of FST and SPT. Corticosterone treatment induces depression-like behavior and anhedonia, manifesting as (B) increased immobility time in the FST and (C) increased sucrose consumption in the SPT. Notably, rotarod training improves both depression-like behaviors and anhedonia under corticosterone-treated conditions. Data are presented as mean ± SEM (*n* = 5–6 rats per group). One-way analysis of variance followed by the least significant difference *post hoc* test was used. CORT: Corticosterone-treated; FST: forced swimming test; N: no rotarod training; SPT: sucrose preference test; T: rotarod training; V: vehicle-treated.

In this study, an animal model to assess depression-like behaviors and symptoms of motor retardation induced by corticosterone treatment was used. Continuous administration of corticosterone over a 14-day period resulted in significant behavioral changes indicative of depression. The results from the forced swimming test (FST) revealed a marked increase in immobility time among the corticosterone-treated animals. Specifically, the corticosterone-only group (CORT_N) demonstrated an immobility time of 185.64 ± 12.25 seconds (*P* < 0.001; **Figure 1B**), indicating heightened despair-like behavior compared to the vehicle-treated controls, which exhibited an immobility time of 101.58 ± 10.19 seconds (V_N). Importantly, the concurrent administration of corticosterone with rotarod training resulted in a significant alleviation of these depression-like behaviors. The corticosterone plus rotarod training group (CORT_T) recorded an immobility time of only 79.90 ± 13.10 seconds in the FST, which was notably lower than that observed in the CORT_N group and comparable to the vehicle-treated controls that underwent rotarod training (V_T: 53.82 ± 24.90 seconds, *P* < 0.001). These results show that corticosterone administration induces significant depression-like behaviors, characterized by increased immobility and reduced sucrose consumption. However, the addition of rotarod training during corticosterone treatment markedly alleviates these symptoms, demonstrating the therapeutic potential of physical activity in managing corticosterone-induced depression-like behaviors and motor retardation in this model.

### Depression indicators

Similarly, the SPT results indicated a normalization of sucrose consumption in the CORT_T group, which showed a preference of 84.14% ± 3.81%, comparable to the vehicle-treated group with rotarod training (V_T: 86.46% ± 2.74%). Further evidence of depression was observed in the SPT, where the corticosterone-treated group (CORT_N) exhibited a significantly reduced sucrose consumption of 52.59% ± 8.13% (*P* = 0.008; **Figure 1C**), suggestive of anhedonia. In contrast, the vehicle-treated controls showed a substantially higher sucrose preference (V_N: 74.33% ± 6.06%). Importantly, the concurrent administration of corticosterone with rotarod training resulted in a significant alleviation of these depression-like behaviors. The corticosterone plus rotarod training group (CORT_T) recorded an immobility time of only 79.90 ± 13.10 seconds in the FST, which was notably lower than that observed in the CORT_N group and comparable to the vehicle-treated controls that underwent rotarod training (V_T: 53.82 ± 24.90 seconds). Similarly, the SPT results indicated a normalization of sucrose consumption in the CORT_T group, which showed a preference of 84.14% ± 3.81%, comparable to the vehicle-treated group with rotarod training (V_T: 86.46% ± 2.74%). These results show that corticosterone administration induces significant depression-like behaviors, characterized by increased immobility and reduced sucrose consumption. However, the addition of rotarod training during corticosterone treatment markedly alleviates these symptoms, demonstrating the therapeutic potential of physical activity in managing corticosterone-induced depression-like behaviors and motor retardation in this model.

### Motor impairments induced by corticosterone

**Figure 2:**
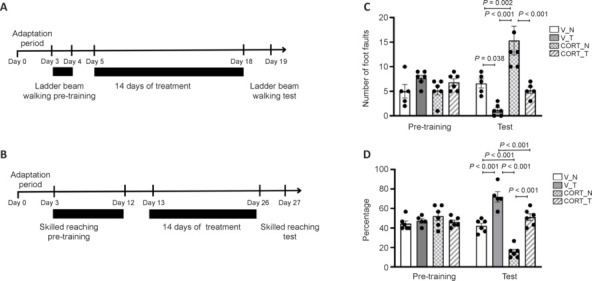
Induction of motor deficits in animals by corticosterone and therapeutic role of rotarod training on animal behaviors. (A, B) The experimental timeline for ladder beam walking and skilled reaching tests, respectively. (C) The increased number of foot faults in the ladder beam walking test. (D) The decreased percentage of success in reaching foot pellets in the skilled reaching test, both indicative of motor deficits in CORT-treated animals. Rotarod training (T) significantly improved motor performances under both vehicle-treated (V) and CORT-treated conditions in both tests. Motor performances, as measured by ladder beam walking and skilled reaching, did not differ significantly between groups prior to treatment (pre-training, C and D). Data are presented as mean ± SEM (*n* = 5–6 rats per group). One-way analysis of variance followed by the least significant difference *post hoc* test was used. CORT: Corticosterone-treated; N: no rotarod training; T: rotarod training; V: vehicle-treated.

The 14-day corticosterone injection protocol not only induced depression-like behaviors in the animals but also led to significant motor impairments in both forelimbs and hindlimbs. Specifically, rats receiving corticosterone treatment showed a considerable reduction in their success rate for retrieving food pellets during the SRT, with a success rate of only 15.56 ± 2.53% (CORT_N) (*P* < 0.001; **Figure 2D**). In addition, hindlimb performance was adversely impacted, as evidenced by an increased number of foot faults observed during the LBWT. The corticosterone-treated rats exhibited 15.53 ± 2.93 foot faults (CORT_N), in stark contrast to the vehicle-treated control groups, which had a success rate of 42.22 ± 2.68% (V_N, *P* < 0.001) for the SRT and 72.0 ± 5.33% (V_T, *P* < 0.001) for the SRT. In the LBWT, vehicle-treated rats demonstrated fewer foot faults, averaging 6.60 ± 2.68 (V_N) and just 1.17 ± 0.48 (V_T) (**Figure 2C**). Notably, implementation of rotarod training showed promising effects in ameliorating these motor deficits associated with corticosterone treatment (*P* = 0.002, *vs*. V_N group and *P* < 0.001, *vs*. V_T group). This was evidenced by an improvement in the success rate for retrieving food pellets, which increased significantly to 51.11% ± 3.51% (CORT_T), as well as a marked reduction in the number of foot faults, which decreased to 5.20 ± 0.66 (CORT_T, *P* < 0.001) under the same conditions. These results highlight the potential of rotarod training to counteract motor impairments induced by corticosterone in this model.

### Effects of rotarod training on neural cell proliferation

The effect of rotarod training on BrdU-positive cell populations in the motor cortex was investigated, particularly in response to corticosterone treatment. BrdU was administered during the final three days of treatment to trace proliferating new cells. The quantification of BrdU-positive cells was conducted across the distinct layers of the motor cortex, specifically layers I, II–III, and V–VI.

### Layer I of the motor cortex: cellular changes and effects of corticosterone and training

**Figure 3:**
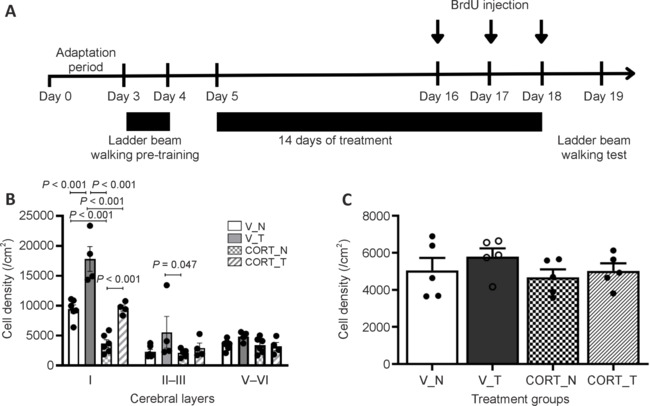
Quantifications of proliferating cells in motor cortex and piriform cortex. (A) The experimental timeline shows when BrdU was administered to track proliferating cells. (B) Corticosterone and rotarod training affected layer I of the motor cortex, with a significant decrease in proliferating cells observed in animals receiving only corticosterone. Rotarod training increased the number of proliferating cells in layer I of the motor cortex under both vehicle-treated and corticosterone-treated conditions. (C) No significant differences were found in the density of BrdU-positive cells in layer I of the piriform cortex among treatment groups. Data are presented as mean ± SEM (*n* = 5–6 rats per group). One-way analysis of variance followed by the least significant difference *post hoc* test was used. CORT: Corticosterone-treated; N: no rotarod training; T: rotarod training; V: vehicle-treated.

Corticosterone treatment resulted in a significant reduction of BrdU-positive cells, most notably in layer I, where the estimated cell count dropped to 3659.76 ± 630.54 cells/cm^2^ (*P* < 0.001; **Figure 3B**). In contrast, rotarod training under corticosterone treatment effectively counteracted this decrease, restoring the cell count in layer I to 9591.89 ± 501.00 cells/cm^2^, which is comparable to the vehicle-treated control group that exhibited 9429 ± 699.72 cells/cm^2^ (*P* < 0.001). Importantly, this protective effect of rotarod training was not observed in the other cortical layers—II–III and V–VI—suggesting a layer-specific response to the training. Moreover, in the vehicle-treated condition without corticosterone, 14 days of rotarod training significantly increased the number of BrdU-positive cells in layer I, reaching a total of 17820.15 ± 2081.01 cells/cm^2^ (*P* < 0.001, *vs*. V_N group). These findings demonstrate that rotarod training not only mitigates the impact of corticosterone on the proliferation of new cells in layer I of the motor cortex but also enhances cell proliferation under normal conditions.

### Region-specific cellular changes: Piriform cortex analysis

The findings indicate that the observed cellular changes are closely related to motor function and are specifically localized to certain brain regions associated with motor activity. Notably, the analysis of BrdU-positive cells in the piriform cortex, a region recognized for its independence from motor control, revealed no significant influence from either corticosterone treatment or rotarod training. The cell counts in the piriform cortex were as follows: in the vehicle non-training group (V_N), there were 5047.16 ± 677.36 cells/cm^2^; in the vehicle training group (V_T), 5793.68 ± 445.03 cells/cm^2^; in the corticosterone non-training group (CORT_N), 4678.65 ± 425.40 cells/cm^2^; and in the corticosterone training group (CORT_T), 5026.28 ± 401.65 cells/cm^2^, as illustrated in **Figure 3C**. These findings emphasize the specificity of the cellular changes to motor-related regions, reinforcing the notion that the motor cortex is likely the primary site affected by corticosterone treatment.

### Cell lineage and marker expression in layer I

**Figure 4:**
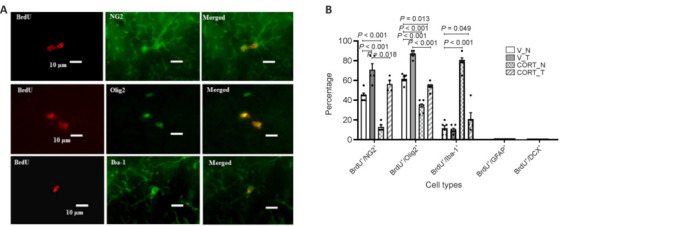
Co-expression percentage of oligodendrocyte-lineage markers with BrdU-positive cells in layer I of the motor cortex. (A) Representative photomicrographs show BrdU-positive cells co-expressed with NG2 marker (yellow) in layer I of the cerebral under different treatments. (B) Most BrdU-positive cells (red) co-expressed with NG2 (green) and Olig2 (green) markers, with a significant difference between the corticosterone-treated group with motor training (CORT_N) and the other three groups. Data are presented as mean ± SEM (*n* = 5–6 rats per group). One-way analysis of variance followed by the least significant difference *post hoc* test was used. CORT: Corticosterone-treated; N: no rotarod training; T: rotarod training; V: vehicle-treated.

In the analysis of layer I of the motor cortex, we found that oligodendrocyte-lineage cells predominantly comprise the proliferating BrdU-positive cells. Through co-labeling with specific lineage markers, we were able to categorize the BrdU-positive cells into various cell types, revealing that the majority expressed oligodendrocyte lineage markers. Importantly, none of the identified BrdU-positive cells were labeled with markers for either neuronal lineage (doublecortin) or astrocyte lineage (glial fibrillary acidic protein), underscoring the oligodendrocyte lineage’s significant contribution to cell proliferation in this region. Corticosterone treatment notably affected the expression of oligodendrocyte precursor cell markers in the BrdU-positive population (**Figure 4A**). Specifically, the percentage of BrdU-positive cells expressing NG2 decreased to 13.0% ± 2%, while the proportion expressing Olig2 dropped to 33.3% ± 2.57%. In contrast, rotarod training had a beneficial effect on the co-expression of oligodendrocyte lineage markers in proliferating BrdU-positive cells, regardless of whether the treatment involved vehicle or corticosterone. The vehicle treatment group (V_T) demonstrated a significantly higher co-expression of NG2 (71.0% ± 6%) and Olig2 (80.0% ± 0.91%), while the corticosterone treatment group (CORT_T) exhibited lower (*P* = 0.018 and *P* < 0.001, respectively), yet still substantial expression levels of NG2 (56.3% ± 3.75%) and Olig2 (50.0% ± 1.96%) (**Figure 4B**). These findings highlight the robust role of oligodendrocyte-lineage cells in layer I of the motor cortex, particularly in the context of rotarod training which appears to enhance their proliferative capacity even under corticosterone treatment.

### Microglial activation and interaction with oligodendrocyte-lineage cells

Moreover, significant findings regarding the co-expression of BrdU-positive cells with specific markers in layer I of the motor cortex were observed. Notably, BrdU-positive cells exhibited co-expression with the microglial marker Iba-1, highlighting a potential interaction between oligodendrocyte-lineage cells and microglia in this region. In animals treated solely with corticosterone (CORT_N), we recorded a substantial increase in the percentage of Iba-1/BrdU double-positive cells, reaching 79.0% ± 1.11%. This value was markedly higher compared to the vehicle control groups, V_N (11.67% ± 2.47%, *P* < 0.001) and V_T (9.17% ± 2.39%, *P* < 0.001). Additionally, the inclusion of rotarod training for animals under corticosterone treatment (CORT_T) resulted in a significant decrease in the percentage of Iba-1/BrdU double-positive cells, dropping to 21.0% ± 1.72% (*P* < 0.001, *vs*. V_N group). These results indicate that corticosterone treatment not only enhances the presence of microglial activity but that rotarod training effectively mitigates this effect under stress conditions (**Figure 4B**).

### Myelin protein expression and the impact of corticosterone and training

**Figure 5:**
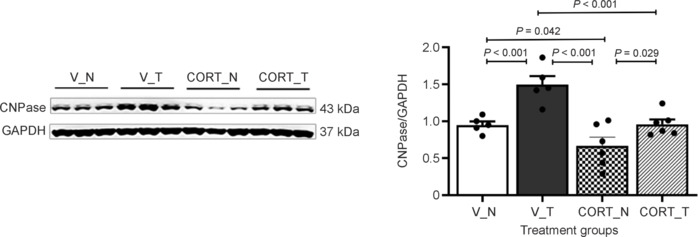
Quantification of myelin-related protein, CNPase, in the motor cortex. Rotarod training increases CNPase expression levels in the motor cortex in rats treated with vehicle or corticosterone. Corticosterone treatment alone significantly alters CNPase expression levels. Data are presented as mean ± SEM (*n* = 5–6 rats per group). One-way analysis of variance followed by the least significant difference *post hoc* test was used. CNPase: 2′,3′-Cyclic nucleotide 3′-phosphodiesterase; CORT: Corticosterone-treated; GAPDH: glyceraldehyde-3-phosphate dehydrogenase; N: no rotarod training; T: rotarod training; V: vehicle-treated.

The results of our study indicate that corticosterone treatment significantly affects the expression of the myelin-related protein CNPase in the motor cortex. Quantitative Western blot analysis revealed that CORT treatment resulted in a marked reduction of CNPase levels, with values of 0.60 ± 0.11, compared to the control condition (CORT_N, P=0.042). In contrast, rotarod training effectively mitigated this reduction, leading to a significant increase in CNPase expression under both vehicle-treated (V_T: 1.45 ± 0.11; *P* < 0.001) and corticosterone-treated (CORT_T: 0.96 ± 0.06) conditions. These findings suggest that while CORT treatment adversely impacts myelin-related protein expression in the motor cortex, rotarod training can counteract this effect, supporting the potential therapeutic role of motor training in managing the consequences of hypercortisolemia (**Figure 5**).

### Activation of BrdU-positive cells by motor activity

**Figure 6:**
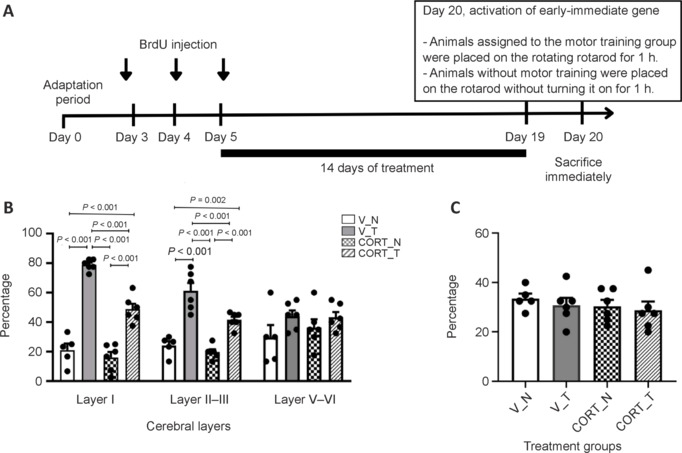
Treatment and BrdU expression per cerebral layer. (A) Treatment schedule and activation of the early-immediate gene (*egr-1*). (B) egr-1 co-expression with BrdU in the motor cortex. (C) Percentage of egr-1/BrdU co-expression in the piriform cortex. Data are presented as mean ± SEM (*n* = 5–6 rats per group). One-way analysis of variance followed by the least significant difference *post hoc* test was used. BrdU: Bromodeoxyuridine; CORT: corticosterone-treated; N: no rotarod training; T: rotarod training; V: vehicle-treated.

The results of the activation of BrdU-positive cells in the motor cortex by motor activities revealed significant findings. Co-labeling of BrdU-positive cells with the immediate-early gene marker egr-1 demonstrated that rotarod training effectively activated these proliferating cells, particularly in layers I–III of the motor cortex. This activation was observed in both vehicle and corticosterone-treated groups when compared to groups that did not undergo motor training. Specifically, the percentage of activated BrdU-positive cells in layer I was higher in the vehicle-treated group (21.0% ± 4.46%) compared to the corticosterone-treated group (16.0% ± 9.18%). In layers II–III, the vehicle-treated group showed a similar trend, with 24.17% ± 2.91% activation, while the corticosterone-treated group registered 19.72% ± 1.88% activation. Furthermore, the level of activation was assessed through the co-expression of BrdU with the egr-1 marker, yielding a clearer understanding of the differential effects of treatment. The vehicle-treated group displayed a notably elevated level of egr-1 expression in layers I–III, with values of 78.75% ± 1.55% in layer I (*P* < 0.001) and 61.25% ± 5.35% in layers II-III (*P* < 0.001). In contrast, the corticosterone-treated group exhibited reduced levels of egr-1 expression, with 48.72% ± 8.95% (*P* < 0.001) in layer I and 61.25% ± 5.35% (*P* < 0.001) in layers II–III. These results indicate that motor activity, particularly through rotarod training, significantly enhances the activation of BrdU-positive cells in the motor cortex, with vehicle treatment leading to a more pronounced activation compared to corticosterone treatment (**Figure 6**).

### Layer V–VI co-expression and regional specificity

The analysis of co-expression percentages in layers V-VI of the motor cortex revealed no significant differences among the four treatment groups: control (V_N) with 29.67% ± 8.37%, treatment (V_T) at 44.17% ± 3.69%, corticosterone-treated control (CORT_N) at 36.25% ± 5.69%, and corticosterone-treated treatment (CORT_T) at 43.19% ± 3.62%. These findings suggest that the observed co-expression levels are consistent across different experimental conditions within this specific cortical layer. Furthermore, these changes appear to be region-specific, as no significant differences were detected in the number of BrdU-positive cells displaying egr-1 immunoreactivity in the piriform cortex across the treatment groups: V_N at 33.5% ± 2.03%, V_T at 30.83% ± 3.00%, CORT_N at 30.42% ± 2.62%, and CORT_T at 29.75% ± 3.58% (**Figure 6C**). Collectively, these results indicate that the treatment-related alterations in co-expression are localized to the motor cortex and do not extend to the piriform cortex.

## Discussion

The findings from this study indicate that corticosterone treatment induces significant depression-like behaviors and motor impairments in an animal model, as evidenced by increased immobility in the forced swimming test and reduced success rates in skilled reaching and ladder beam walking tests. The concurrent implementation of rotarod training notably alleviated these behavioral symptoms, demonstrating its therapeutic potential in mitigating corticosterone-induced effects. Specifically, rotarod training improved motor performance and normalized sucrose consumption, suggesting that physical activity not only enhances motor function but also addresses the anhedonia associated with depressive states.

Motor impairment, particularly in the context of depression, can be improved through targeted motor training interventions, such as rotarod training. This type of training not only enhances motor performance but also has beneficial effects on oligodendrocyte lineage cells (OLs) in the motor cortex. The rotarod provides standardized daily motor training and allows for social housing, which helps mitigate stress that may negatively impact motor function (Hamm et al., 1994; Holschneider et al., 2007; Aguiar et al., 2009; Seo et al., 2010). Studies have demonstrated that rotarod training significantly increases synaptogenesis and brain-derived neurotrophic factor (BDNF) levels, as well as elevates the number of c-fos positive cells in the motor cortex (Kleim et al., 1996; Jones et al., 1999; Milgram et al., 2006). Furthermore, rotarod training has been shown to improve motor deficits induced by stress and promote the population of proliferating OLs in both normal and stressed conditions, as indicated by increases in BrdU^+^/NG2^+^ and BrdU^+^/Olig2^+^ cells (Eugenin von Bernhardi and Dimou, 2022). Additionally, rotarod training appears to mitigate the suppression of oligodendrocyte precursor cell activation that occurs with stress, providing evidence for the role of motor training in fostering neurophysiological changes that contribute to the improvement of motor performance (Eugenin von Bernhardi and Dimou, 2022). It suggests that the therapeutic effect of motor training may be linked to enhanced oligodendrogenesis in the motor cortex, along with potential improvements in myelination processes that facilitate motor skill acquisition (McKenzie et al., 2014). In summary, rotarod training aids in reversing psychomotor retardation associated with depression by promoting the health and proliferation of oligodendrocyte-lineage cells and enhancing myelination in the motor cortex. This approach could pave the way for developing novel strategies to ameliorate adverse symptoms of depression related to motor function.

Furthermore, the study revealed layer-specific effects of corticosterone treatment and rotarod training on oligodendrocyte lineage cells within the motor cortex. Corticosterone decreased the proliferation of BrdU-positive oligodendrocyte precursor cells, particularly in layer I, while rotarod training effectively restored these populations and even elevated them under normal conditions. These results highlight the critical role of oligodendrocyte lineage cells in the context of both motor function and neuropsychiatric disorders, reinforcing the notion that motor activity can positively influence neuroplasticity in this specific cortical layer. Recent studies have highlighted changes in OLs within the central nervous system, which have been linked to depressive symptoms. While previous research primarily focused on emotional and cognitive regions, there has been limited evidence addressing changes in OLs within motor-related areas, such as the motor cortex, in relation to psychomotor retardation symptoms observed in depression. In alignment with findings such as those by Alonso (2000), who demonstrated that chronic corticosterone treatment inhibits the proliferation of OPCs in the rodent brain, our study specifically examined the effects within the motor cortex. We found that 14 days of corticosterone treatment resulted in a significant decrease in the number of proliferating OLs, specifically BrdU^+^/NG2^+^ and BrdU^+^/Olig2^+^ cells, which correlated with motor deficits exhibited in skilled reaching and ladder beam walking tests. Further investigation revealed that rotarod training not only mitigates motor deficits due to stress, but also modulates the oligodendrocyte lineage cell population within the motor cortex. Prior research by Eugenin von Bernhardi and Dimou (2022) indicated that physical activity could promote oligodendrogenesis in the motor cortex under normal conditions (Eugenin von Bernhardi and Dimou, 2022). Our findings extended this notion by showing that rotarod training stimulates both BrdU^+^/NG2^+^ and BrdU^+^/Olig2^+^ cells in both normal and corticosterone-treated conditions. This training alleviated the suppression of oligodendrocyte lineage cells during motor tasks that were induced by corticosterone treatment. The findings of this study revealed that significant changes in proliferating OLs occurred only within layer I of the motor cortex when compared to other cortical layers, under both corticosterone treatment and rotarod training. This finding is consistent with the research by Eugenin von Bernhardi and Dimou (2022) that noted physical activity’s impact across all layers. The differences may be attributed to the varying BrdU administration paradigms, with our study focusing on the last 3 days to assess proliferating OLs specifically. By employing an immediate early gene marker, *egr-1*, we visualized the co-localization of BrdU^+^ cells with oligodendrocytes and noted that corticosterone treatment significantly suppressed cell activation. However, in the rotarod-trained group, this suppression was alleviated during motor tasks.

Cortical layer I’s role in neuropsychiatric disorders is underscored by its extensive neural projections, particularly as it receives inputs from dopaminergic and serotonergic pathways critical to motor task processing and mood regulation (Gaspar et al., 1991; Banasr et al., 2007). Our findings demonstrate that corticosterone treatment significantly alters the population of OLs in layer I of the motor cortex, suggesting that this layer may be crucial for exploring psychomotor retardation symptoms associated with depression. However, further research is required to elucidate how the treatment processes modulate the function of OLs over time. In conclusion, the therapeutic approach of rotarod training appears to reverse psychomotor retardation symptoms associated with depression by enhancing oligodendrocyte-lineage cells and promoting myelination in the motor cortex. The findings suggest that layer I—a site impacted adversely by stress and directly associated with motor impairments—may play a pivotal role in understanding the neural mechanisms underlying psychomotor retardation symptoms. Further investigation into the neural circuitry within this layer could lead to novel strategies for alleviating the adverse symptoms of depression.

The findings underscore the importance of layer I in the motor cortex and its potential link to psychomotor retardation observed in depression. Alterations in myelin-related proteins, such as CNPase, further suggest that stress impacts myelination, with rotarod training helping to preserve myelin expression. CNPase accounts for 4% of total myelin protein in the central nervous system and is the most abundant non-compact myelin protein (Raasakka et al., 2015). The absence of CNPase in oligodendrocytes is thought to hinder a crucial glial trophic support mechanism for axons (Verrier et al., 2013). Overall, this study proposes that rotarod training may serve as an effective intervention for reversing depression-related motor deficits by enhancing oligodendrocyte function and promoting myelination in the motor cortex, particularly in layer I, which may reveal new avenues for therapeutic strategies in managing depression-associated symptoms.

Adaptive myelination is the dynamic process by which OLs regulate the formation and maintenance of myelin sheaths around axons in response to neural activity and environmental demands, optimizing signal transmission and neural circuit efficiency (Gibson et al., 2014; McKenzie et al., 2014). In this process, OLs adjust the thickness of myelin sheaths around axons in response to neural activity, and CNPase plays a crucial role in this process by facilitating the synthesis and maintenance of myelin, which is essential for optimal motor function (Gibson et al., 2014; McKenzie et al., 2014). Recent studies indicate that stress significantly impacts the population of OLs in the motor cortex, particularly through the effect of corticosterone treatment, which mimics a hypercortisolemia state observed in depressed patients. Notably, Western blot findings indicated a stress-induced reduction in CNPase—a myelin protein—by approximately half in the motor cortex (McKenzie et al., 2014). Furthermore, rotarod training was shown to maintain the expression of this myelin protein at levels comparable to those in the control group under stress conditions, suggesting that target OLs may contribute to the adult myelination process as it relates to motor performance. The findings imply that stress adversely affects myelination and that rotarod training could help in mitigating these effects. In summary, the therapeutic approach of rotarod training may reverse psychomotor retardation associated with depression by enhancing oligodendrocyte-lineage cells and promoting myelination in the motor cortex, further implicating CNPase in these processes.

This study has some limitations that should be noted. First, as an animal study, the findings may not fully generalize to humans with depression, and translational research is needed to confirm these mechanisms in clinical populations. Second, the study focused primarily on specific cortical layers and regions, so the broader neural circuitry involved in psychomotor retardation was not fully explored.

In summary, we propose that the therapeutic approach of rotarod training may reverse psychomotor retardation associated with depression by enhancing oligodendrocyte-lineage cells and promoting myelination in the motor cortex. Our findings suggest that cortical layer I—a site where oligodendrocytes are adversely affected by stress and associated with motor impairments—may be closely related to psychomotor retardation symptoms. Further elucidation of the neural circuitry involved in this layer could facilitate the development of novel strategies aimed at ameliorating adverse symptoms of depression.

## Data Availability

*All relevant data are within this paper*.
